# *Drosophila* poly suggests a novel role for the Elongator complex in insulin receptor–target of rapamycin signalling

**DOI:** 10.1098/rsob.110031

**Published:** 2012-01

**Authors:** Ekin Bolukbasi, Sharron Vass, Neville Cobbe, Bryce Nelson, Victor Simossis, Donald R. Dunbar, Margarete M. S. Heck

**Affiliations:** University of Edinburgh, Queen's Medical Research Institute, University/BHF Centre for Cardiovascular Science, 47 Little France Crescent, Edinburgh EH16 4TJ, UK

**Keywords:** cell growth, nucleus, signalling, insulin

## Abstract

Multi-cellular organisms need to successfully link cell growth and metabolism to environmental cues during development. Insulin receptor–target of rapamycin (InR–TOR) signalling is a highly conserved pathway that mediates this link. Herein, we describe *poly*, an essential gene in *Drosophila* that mediates InR–TOR signalling. Loss of *poly* results in lethality at the third instar larval stage, but only after a stage of extreme larval longevity. Analysis in *Drosophila* demonstrates that Poly and InR interact and that *poly* mutants show an overall decrease in InR–TOR signalling, as evidenced by decreased phosphorylation of Akt, S6K and 4E-BP. Metabolism is altered in *poly* mutants, as revealed by microarray expression analysis and a decreased triglyceride : protein ratio in mutant animals. Intriguingly, the cellular distribution of Poly is dependent on insulin stimulation in both *Drosophila* and human cells, moving to the nucleus with insulin treatment, consistent with a role in InR–TOR signalling. Together, these data reveal that Poly is a novel, conserved (from flies to humans) mediator of InR signalling that promotes an increase in cell growth and metabolism. Furthermore, homology to small subunits of Elongator demonstrates a novel, unexpected role for this complex in insulin signalling.

## 2. Introduction

Multi-cellular organisms have evolved mechanisms to link cellular metabolism and growth to external environmental cues, such as nutrient and growth factor levels, in order to survive and adapt to fluctuations in the availability of these factors. The InR–TOR pathway is one of the key regulators of cellular energy homeostasis and growth [1], and this signalling pathway is evolutionarily conserved among metazoa [1,2]. Thus, studies carried out using *Drosophila* as a model system have played a major role in expanding our understanding of the mechanisms, as well as downstream consequences, of signalling via this pathway [3–5]. In humans, aberrations of InR–TOR signalling lead to various metabolic syndromes, including diabetes and obesity, as well as to the development of various types of cancers [6].

A cascade of phosphorylation events mediates signalling through the InR–TOR pathway. The binding of insulin to the InR leads to the phosphorylation of insulin receptor substrate (IRS) by the InR. IRS acts as a recruitment site for phosphatidylinositol 3-kinase (PI3K), which catalyses the conversion of phosphatidylinositol (4,5)-bisphosphate (PIP_2_) into phosphatidylinositol (3,4,5)-trisphosphate (PIP_3_) at the cell membrane. PIP_3_ in turn recruits PDK1 and Akt to the membrane, where PDK1 phosphorylates and activates Akt. Phosphorylated Akt signals inhibit the tuberous sclerosis complex (TSC, Tsc1–Tsc2) [7–9]. When TSC is inhibited, the small GTPase Rheb becomes active [10]. This then activates TOR, integrating TOR into the insulin signalling process.

TOR is a component of two different complexes: TORC1 and TORC2. Activation of TORC1 has various downstream effects contributing to an increase in cell growth and proliferation. For example, TORC1 directly phosphorylates S6K and 4E-BP, resulting in an increase in ribosome biogenesis and m7G cap-dependent translation [1]. In addition, TORC1 activation inhibits autophagy [11]. A negative feedback loop signals back to the IRS through S6K, ensuring attenuation of TOR signalling above a certain level [12]. The TORC2 complex phosphorylates and activates Akt kinase [13], resulting in the phosphorylation of the forkhead-like transcription factor FoxO. Phosphorylated FoxO is excluded from the nucleus, precluding the transcription of FoxO target genes [14–16].

A critical consequence of the activation of InR–TOR signalling is the inhibition of autophagy: a cellular response to starvation in which components of the cytoplasm are engulfed in small double-membrane-enclosed vesicles. The contents of these vesicles are degraded by the autophagic machinery, and breakdown products then serve as a nutrient source for the cell until more nutrients become available in the environment. Alterations to autophagy have been found in cancer and neurodegenerative diseases [17,18].

Herein, we report the identification of Poly as a novel mediator of the InR–TOR signalling pathway. *poly* is an essential gene in *Drosophila* that was mutated in a P-element transposon mutagenesis screen [19]. Crucially, the gene product is conserved in higher eukaryotes, including humans, showing homology to the ELP6 subunit of the Elongator complex. Loss of *poly* function results in lethality at the late larval stage, but only after extreme larval longevity. Many intriguing phenotypic features are observed in larvae lacking *poly*, including abnormal nuclear morphology in neuroblasts and the development of large melanotic masses in third instar larvae. We have combined genetic, biochemical and bioinformatic approaches to functionally characterize *poly*. Our data reveal that *poly* is a novel mediator of InR–TOR signalling and that loss of *poly* results in downregulation of a number of components of the InR–TOR pathway. We therefore propose that the wild-type Poly protein is a positive regulator of cell growth and metabolism.

## 3. Results

### 3.1. Characterization of the poly mutant phenotype

**Figure 1.**
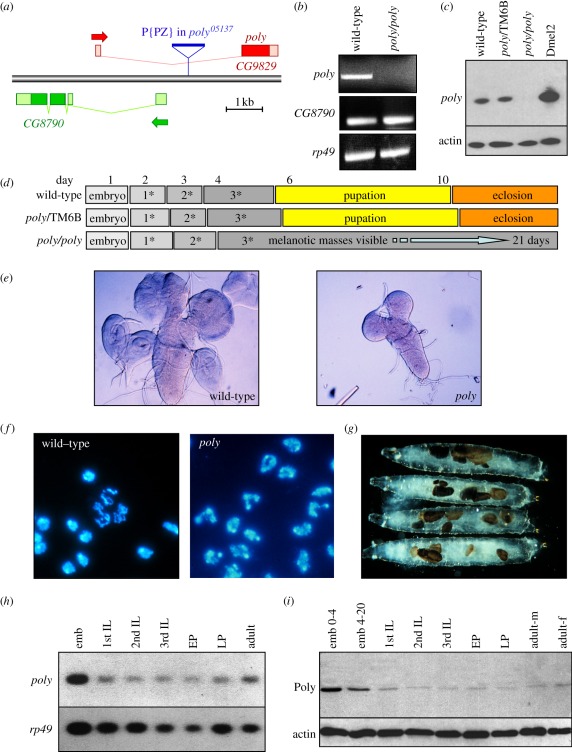
Characterization of the *poly* gene and mutant phenotype. (*a*) Gene map showing the P-element insertion in the first intron of *poly*. (*b*) RT-PCR from wild-type and homozygous *poly* third instar larvae demonstrating the absence of Poly in *poly* mutant animals. The level of CG8790 is unaffected in *poly* mutants. RP49 serves as a control. (*c*) Immunoblotting of extracts from wild-type, heterozygous and homozygous *poly* third instar larvae demonstrating the absence of Poly in *poly* mutant animals. Poly is also present in Dmel2-cultured *Drosophila* cells. (*d*) Developmental timing of wild-type, heterozygous and homozygous *poly* animals. (*e*) Third instar larval brain and imaginal discs dissected from wild-type and *poly* animals. Note the reduced brain size and absence of imaginal discs in the homozygous *poly* mutant. (*f*) DAPI staining of wild-type and *poly* larval neuroblasts. Note the abnormally shaped nuclei in the homozygous *poly* mutant. (*g*) Homozygous *poly* larvae develop melanotic masses during the third larval instar, which increase in number with time. (*h*,*i*) Expression analysis of *poly* mRNA and protein throughout development. Northern and immunoblotting of wild-type extracts at specific developmental stages showing Poly mRNA and protein expression levels. emb, embryo (0–4, 4–20 = age in hours); IL, instar larvae (1st, 2nd and 3rd); EP, early pupae; LP, late pupae; adult-m, adult males; adult-f, adult females.

The *poly* mutation was isolated in a P-element mutagenesis screen that aimed to generate a large collection of single P-element-induced mutations in *Drosophila* [19]. The P-element insertion that led to the lethal *poly^05137^* allele was mapped to the single intron of the CG9829 gene, localizing to 87E7-8 on the third chromosome (figure 1*a*). The *poly^05137^* insertion led to an absence of *poly* mRNA as assessed by Northern blotting (not shown), reverse transcriptase–polymerase chain reaction (RT-PCR; figure 1*b*) and Poly protein as revealed by immunoblotting (figure 1*c*). RT-PCR verified that expression of the overlapping CG8790 gene was not affected by the P-element insertion in the *poly^05137^* allele (figure 1*b*). Two independent experiments additionally corroborate lesion of the CG9829 gene as being responsible for the mutant phenotype: (i) excision of the P-element following genetic exposure to transposase completely reverted the mutant phenotype, and (ii) successful rescue of *poly^05137^* lethality was achieved by using a hs-Gal4 driver to direct expression of a *UAS-poly* transgene during larval development.

Mutation of *poly* results in pleiotropic effects manifesting as a particularly striking phenotype. *poly* mutants appear to progress normally through embryogenesis, but larval development proceeds much more slowly. While the normal generation time is 10 days at 25°C, *poly* mutant larvae exhibit extreme third instar longevity—up to 21 days—before dying without pupation (figure 1*d*). When homozygous mutant larvae were examined, the morphology of many tissues appeared abnormal: the brain, ring gland, salivary glands and imaginal discs were reduced in size compared with heterozygous siblings and wild-type animals, suggesting cell growth and/or proliferation defects (figure 1*e*). Mutant larval neuroblasts were characterized by abnormally shaped nuclei, though mitotic figures, evident even in 20 day old mutant larvae, were normal in appearance. These lobulated nuclei resembled the nuclei of mammalian polymorphonuclear leucocytes, thus suggesting the name *poly* (figure 1*f*). During the lengthened third instar larval phase, melanotic masses appeared in the haemolymph of *poly* mutants, increasing in size and number with time (figure 1*g*). A second hypomorphic allele of *poly* has recently been reported to result in abnormal nurse-cell chromosome dispersal and oocyte polarity in the *Drosophila* germline, possibly owing to a proposed interaction with an mRNP complex involved in similar processes [20].

Expression analysis of *poly* revealed high levels of mRNA and protein in the first 4 h of embryogenesis, suggestive of maternal loading of the mRNA and possibly protein into oocytes (figure 1*h*,*i*). While the level of Poly decreased during the remainder of embryogenesis, the level was still higher than in later stages of development. Overall, *poly* was expressed throughout development (figure 1*h*,*i*).

### 3.2. Poly is conserved among higher eukaryotes and resembles the Elp6 subunit of the Elongator complex

**Figure 2.**
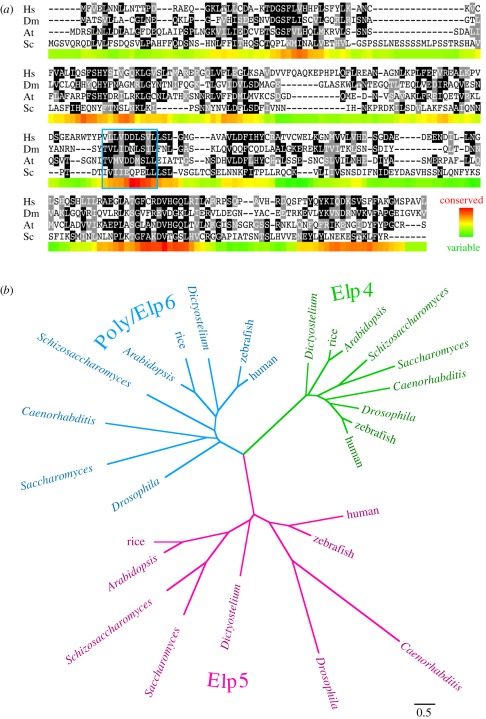
Alignment and phylogenetic analysis of Poly. (*a*) Alignment showing Poly homologues in human (Hs; NP_001026873), *Drosophila melanogaster* (Dm; AAF40432), *Arabidopsis thaliana* (At; NP_567351) and *Saccharomyces cerevisiae* (Sc; NP_014043), with shading of identical and similar amino acids as rendered by the BOXSHADE program. The coloured band underneath each section of the alignment indicates sequence conservation according to the PAM250 matrix, averaged over a sliding window of 10 amino acids. The location of the Walker B motif (ordinarily associated with ATP binding) is indicated by a blue box, although no Walker A (P-loop) motif required for ATP hydrolysis is similarly conserved. (b) Phylogenetic tree showing the relationship between Poly, identified as the *Drosophila* orthologue of Elp6, and homologous sequences. The scale bar indicates distance according to the WAG substitution matrix.

The *Drosophila poly* gene encodes a 251-amino-acid-long protein, lacking any motifs suggestive of specific function. Sequence similarity searches revealed that Poly is conserved among higher eukaryotes and, importantly, has a human homologue (figure 2*a*). The apparent orthologue of Poly in *Saccharomyces cerevisiae* is the Elp6 protein, part of the 6-subunit Elongator complex that, in association with the RNA polymerase II holoenzyme, is responsible for transcriptional elongation [21,22]. The Elongator holocomplex is conserved in composition from yeast to humans [23,24], with acetylation activity contributed by the Elp3 catalytic subunit [25]. Intriguingly, acetylation is directed to different substrates, depending on where in the cell the complex is (e.g. tubulin in the cytoplasm, and histone in the nucleus) [26,27]. Elongator has additionally been linked to translational control through tRNA modification in the cytoplasm. To date, only the Elp3 subunit has been studied in *Drosophila,* where the mutant phenotype has recently been reported to be remarkably similar to that described herein [28].

Elp6 in budding yeast is part of an Elongator subcomplex that also contains the Elp4 and Elp5 proteins [21,22], which share sufficient homology to be aligned with one another (and can also be identified in flies and humans). Consistent with the previous identification of Elp4 and Elp6 proteins as paralogues [29], our resulting phylogeny implies that each of the three different Elongator subunits probably arose from common gene duplication events during eukaryotic evolution (figure 2*b*; electronic supplementary material, figure S1). Thus, Poly is an evolutionarily conserved protein that exhibits homology to the yeast Elp6 Elongator subcomplex component.

### 3.3. Identification of protein interactors of Poly

**Figure 3.**
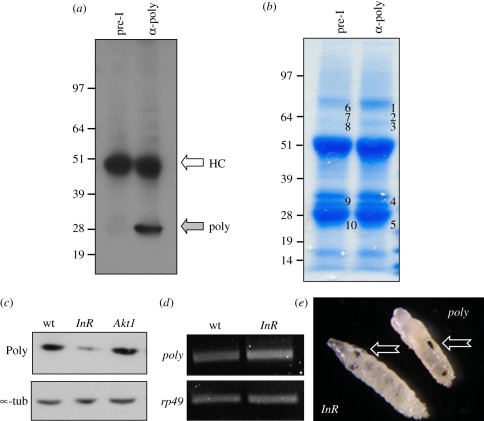
Poly and the insulin receptor physically interact. (*a*) An antibody generated to recombinant Poly immunoprecipitates Poly from wild-type *Drosophila* third instar larval extracts. HC represents the heavy chain recognized by the secondary antibody during detection. Poly migrates at its predicted size of 28 kDa. (*b*) Poly was immunoprecipitated from 0–5 h wild-type embryos using Poly antibodies and compared with an identical sample immunoprecipitated with pre-immune serum. Bands numbered 1–5 in the immunoprecipitate lane and their corresponding bands in the pre-immune lane (numbered 6–10) were excised from the Coomassie-stained gel and analysed by mass spectrometry. (*c*) Immunoblotting using the Poly antibody was carried out on wild-type, *InR^05545^* and *Akt1^04226^* larval extracts*.* (*d*) RT-PCR was performed using primers specific for *poly* and *rp49* on RNA extracted from wild-type and *InR^05545^* larvae. (*e*) Melanotic masses (arrows) observed in *InR^05545^* and *poly* mutant larvae.

As the *poly* gene appeared to be a ‘pioneer’, we adopted an unbiased approach to determine the pathways and processes in which Poly might be involved. In order to identify proteins interacting physically with Poly, immunoprecipitation with an antibody generated against recombinant Poly was performed using 0–5 h *Drosophila* embryo extracts (figure 3*a*,*b*). Poly can only be immunoprecipitated with immune serum, and only from wild-type extracts. Samples were analysed using tandem mass spectrometry [30,31]. Immunoprecipitates with pre-immune serum served as the control. Among the five Poly-binding proteins identified with significant scores, InR scored highest and was represented by numerous peptides in two distinct bands (table 1). This result suggests that Poly and the InR might coexist within a complex, possibly playing a role in the InR signalling pathway.

**Table 1.** Mass spectrometry identifies the insulin receptor as a physical interactor of Poly. Analysis of pre-immune and immunoprecipitation samples by tandem mass spectrometry identified interacting proteins specifically found in the immune-precipitate sample. Insulin receptor was identified in bands 2 and 3, with the highest scores. ‘exp score’ quantifies on a log scale (base 10) the expectation that the hit was achieved by chance, calculated using the program X!Tandem.

band	exp score	unique/total peptides	protein
1	−3	1/1	ATP-dependent RNA helicase (Rm62)
2	−13	4/7	insulin-like receptor
3	−13	4/7	insulin-like receptor
4	−5.3	3/3	cadherin
5	−2.5	2/2	transient receptor potential cation channel subfamily A (TrpA1)

### 3.4. Poly is decreased in insulin receptor mutants

Considering the physical interaction of Poly with InR, we assessed the level of Poly in various InR signalling mutants. Strikingly, the level of Poly was dramatically decreased in *InR^05545^* mutant larvae, whereas the level of Poly in *Akt1* mutant larvae was unaffected (Akt1 is situated downstream of InR; figure 3*c*). The level of *poly* mRNA was unchanged in the *InR* mutant, suggesting that the difference in protein level might be due to instability or decreased protein synthesis of Poly in the absence of InR (figure 3*d*).

Further evidence for a connection between Poly and the InR was provided by phenotypic analysis of *InR^05545^* mutant larvae (figure 3*e*). While this allele was reported previously to be embryonic lethal [32], we noticed that a small number of homozygous *InR^05545^* animals reached the third instar larval stage. Strikingly, the phenotype of these *InR* mutant larvae resembled that observed in *poly* larvae with increased larval lifespan and development of melanotic masses (figure 3*e*). These observations demonstrate that loss of InR leads to a decrease in Poly and that *InR^05545^* mutants exhibit phenotypic features similar to those of *poly* mutant larvae.

### 3.5. Poly mutation results in decreased signalling activity downstream of the insulin receptor

Based on the results presented so far, we hypothesized that there should be genetic interactions between *poly* and components of the InR signalling pathway. Consequences of such interactions could be revealed by the state of downstream effectors of the InR pathway.

**Figure 4.**
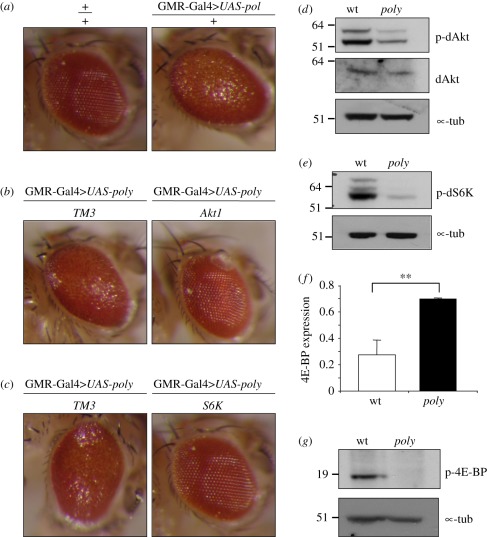
Phosphorylation levels of Akt and S6K are decreased in *poly* larvae. (*a*–*c*) Images showing *Drosophila* compound eyes. (*a*) A rough eye phenotype is caused by *UAS-poly* overexpression under the control of *GMR-Gal4* driver. (*b,c*) The rough eye phenotype caused by *UAS-poly* overexpression under the control of *GMR-Gal4* driver is suppressed by *Akt1^04226^* and *S6K^07084^* mutations. TM3 represents the balancer chromosome carried by control sibling progeny. (*d,e*) Immunoblotting of third instar larval extracts using phospho-specific antibodies to dAkt and dS6K reveals decreased phosphorylation of both kinases in *poly* larval extracts. (*f*) d4E-BP transcript levels were assessed by real-time qPCR on RNA isolated from wild-type and *poly* mutant larvae. d4E-BP levels were normalized to Actin5C levels. The error bars derive from reactions performed on biological triplicate samples. The double asterisk represents significant difference (*p* < 0.01) in 4E-BP expression levels between wild-type and *poly* larvae. (*g*) Immunoblotting of third instar larval extracts showing decreased phosphorylation of d4E-BP in *poly* compared with wild-type larvae.

We therefore over-expressed *poly* in adult fly eyes, using a *GMR-Gal4* construct to drive the expression of a *UAS-poly* transgene [33]. At 27°C, this led to a rough eye (disorganized ommatidia) phenotype (figure 4*a*). Disruption of InR–TOR signalling through mutation of either *dAkt1* or *dS6K* led to a striking suppression of the *poly*-induced rough eye phenotype (figure 4*b*,*c*), suggesting that an intact InR–TOR signalling cascade was required for Poly to exert its effects. These experiments also critically demonstrate the cell autonomous importance of Poly to insulin signalling.

We hypothesized that if *dAkt* and *dS6K* mutants can act as suppressors of the *poly* over-expression phenotype, the activity of these kinases might be altered in *poly* mutant animals. We therefore probed early third instar larval extracts with antibodies that recognize specifically the phosphorylated (active) forms of dAkt and dS6K. Indeed, phosphorylation of both dAkt and dS6K was decreased upon mutation of *poly* (figure 4*d*,*e*). These data reveal that the activity of both dAkt and dS6K kinases was decreased in *poly* mutant animals. In addition, the level of p-Akt at the tissue level, examined by immunofluorescence of whole mount third instar larval brains, additionally corroborates this decrease (electronic supplementary material, figure S2).

One crucial downstream effector of TOR is 4E-BP, the translation initiation factor eIF-4E-binding protein. Phosphorylation of 4E-BP by TOR leads to its dissociation from the m7G-cap-binding protein eIF-4E, thereby allowing activation of cap-dependent translation with consequent positive effects on cell growth [34]. Disruption of InR–TOR signalling causes inhibition of cap-dependent translation as a decrease in 4E-BP phosphorylation results in its binding to eIF-4E [14]. Disruption of signalling also causes increased transcription of 4E-BP owing to increased FoxO activity. Consistent with a decrease in InR–TOR signalling, the level of d4E-BP transcript was almost threefold greater in *poly* mutant larvae compared with control animals (figure 4*f*). In addition, immunoblotting revealed that d4E-BP was hypo-phosphorylated in *poly* mutant extracts compared with wild-type extracts (figure 4*g*). Taken together, these results are consistent with a decrease in cap-dependent translation upon loss of Poly function.

### 3.6. Autophagy is constitutively active in the fat body of poly larvae

A critical consequence of the activation of InR–TOR signalling is the inhibition of autophagy. *Drosophila* undergoes developmentally programmed autophagy at defined times to facilitate tissue remodelling during metamorphosis [35–38]. On the other hand, starvation-induced autophagy (in response to nutrient deprivation) takes place specifically in the larval fat body [11]. As the larval fat body serves a similar function to the vertebrate liver by acting as a nutrient storage organ, it has been commonly used to examine autophagy during both starvation-induced and developmentally regulated autophagy [11,39,40]. While the fat body from fed wild-type larvae does not normally exhibit autophagy, autophagy is evident within a short period of amino acid starvation.

**Figure 5.**
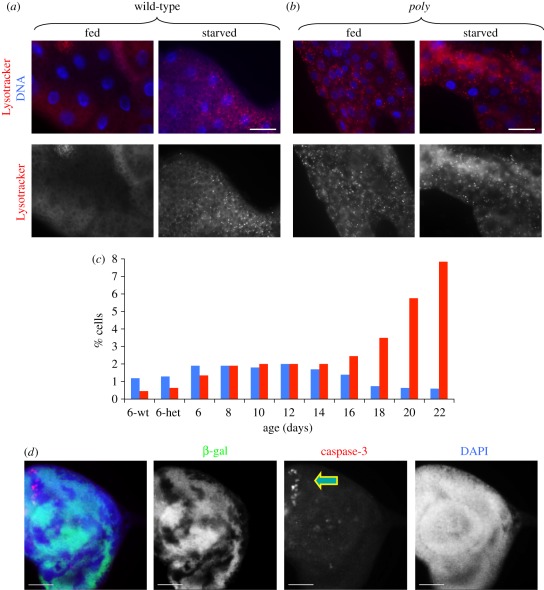
*poly* mutations leads to constitutive autophagy and increased cell death. (*a,b*) Lysotracker (an acidic component-specific fluorescent dye) was used to monitor the state of autophagy in early third instar larval fat body. Autophagy was indicated by a punctate Lysotracker appearance. Live fat body tissues were stained with Lysotracker (red) and Hoechst 33342 to label DNA (blue). (*a*) In wild-type larval fat body, no autophagy is evident. As indicated by Lysotracker positive bright spots, autophagy is active upon 4 h amino acid starvation in 20 per cent sucrose solution. (*b*) Lysotracker puncta can be detected in fed *poly* fat body, as well as in starved *poly* fat body. (*a*,*b*) Scale bar, 50 µm. (*c*) Quantitation of mitosis and apoptosis in larval neuroblasts over development. While mitotic activity decreases, apoptosis increases during the extended larval stage of *poly* larva. Blue bars, p∼H3; red bars, TUNEL. (*d*) Images showing imaginal disc dissected from third instar larva of the genotype *eyFLP; FRT82B*β*-Gal/FRT82Bpoly^05137^*. Immunostaining for cleaved caspase-3 (red), β-galactosidase (green) and DNA (blue) was performed. *poly* mutant clones are indicated by the lack of β-Gal staining. Scale bar, 30 µm.

TOR directly inhibits starvation-induced autophagy, while components of InR signalling (such as InR and Akt, acting upstream of TOR) also behave as negative regulators of autophagy. We hypothesized that if *poly* acts in InR signalling, the state of autophagy as visualized with Lysotracker staining may be altered in the *poly* mutant fat body compared with wild-type fat body. As anticipated, no Lysotracker puncta were observed in fed wild-type early third instar fat body (figure 5*a*, fed). However, Lysotracker puncta became apparent following a 4 h amino acid starvation, owing to the activation of autophagy (figure 5*a*, starved). Strikingly, Lysotracker puncta were abundant even in fed *poly* fat bodies, demonstrating that autophagy was constitutively active in the fat body of *poly* mutants (figure 5*b*, compare fed with starved).

### 3.7. Poly loss of function leads to an increase in apoptotic cell death

Autophagy and apoptosis are closely related, and increased levels of autophagy can lead to apoptosis [39,41]. For example, increased autophagy resulting from the clonal over-expression of *Atg1* in the wing disc resulted in elevated apoptosis, as evidenced by the appearance of cleaved caspase-3 in these clones [39].

Because loss of *poly* led to constitutive activation of autophagy in the fat body, we investigated whether the loss of *poly* also resulted in increased apoptosis. Indeed, cell death increased dramatically in third instar larval neuroblasts as *poly* larvae aged. This was readily seen when larval neuroblasts were stained for pS10-histone H3 and TUNEL to detect mitosis and apoptosis, respectively (figure 5*c*). Mitotic figures, though rare at the latest stages, were still normal in appearance (data not shown). As the generation of mosaic imaginal discs allows the side-by-side comparison of wild-type versus mutant cells, we generated *poly* loss-of-function clones in imaginal discs. Staining of mosaic discs revealed increased levels of cleaved caspase-3 in *poly* mutant clones, which were discernible by the lack of ß-galactosidase (figure 5*d*). Cleaved caspase-3 was not detectable in the adjacent wild-type cells.

Thus, several independent lines of evidence demonstrate that loss of *poly* leads to an activation of autophagy coupled with an increase in apoptotic cell death.

### 3.8. Metabolism is affected in poly mutant larvae

*Drosophila* is frequently used to study metabolic regulation as fruitflies share the majority of metabolic functions with vertebrates [42]. The larval fat body is the main organ for regulation of energy homeostasis as excess energy is stored in the form of glycogen and triglycerides (TAGs; lipids). Activation of signalling through the InR pathway promotes both anabolic metabolism and the storage of nutrients such as TAGs [43]. This is important for development, as the breakdown of larval fat body and consequent release of energy facilitates *Drosophila* metamorphosis.

**Figure 6.**
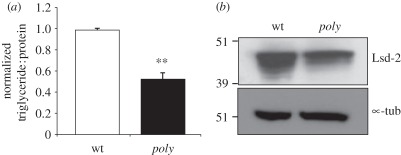
*poly* mutant larvae are characterized by reduced levels of TAGs and Lsd-2. (*a*) TAG levels were assessed in wild-type and *poly* mutant third instar larvae. The total TAG level was normalized to the total protein level. Error bars derive from the standard deviation of three independent experiments. The unpaired two-tailed *t*-test was used to compare the data from wild-type and *poly* larvae. The double asterisk represents a significant difference (*p* < 0.01) in TAG : protein ratio between control and *poly* mutant larvae. (*b*) Immunoblotting was carried out on wild-type and *poly* mutant larval extracts with an antibody specifically recognizing Lsd2 protein.

In order to identify genes that were differentially expressed in *poly* mutant larvae, we carried out a microarray analysis. Among the list of 106 genes downregulated in *poly* mutants, functional enrichment group analysis identified a strong enrichment in gene ontology (GO) terms belonging to metabolic processes, suggesting that metabolism might be affected in *poly* mutants (table 2). We therefore investigated whether the decrease in InR–TOR activity was also manifest at the metabolic level in the storage of TAGs in *poly* mutants. TAG levels were normalized to total protein to give an accurate quantification of lipids per unit mass in wild-type and *poly* mutant larval extracts. Consistent with a decrease in InR–TOR signalling, we found the TAG : protein ratio to be half that detected in wild-type larvae (figure 6*a*).

**Table 2.** Microarray analysis revealed that several metabolic functions were among the genes downregulated in poly mutants. DAVID (the Database for Annotation, Visualization and Integrated Discovery) functional enrichment group analysis of the list of differentially expressed genes identified enrichment in gene ontology (GO) terms belonging to metabolic processes as among those genes downregulated in *poly* mutants. The data were analysed by Limma (see §5.10) and were for four biological replicates. Genes were selected for functional/pathway analysis if the adjusted (corrected for multiple testing) *p*-value was less than 0.05. Log (base 2) fold changes are given (log_2_FC). *‘rep’* indicates reported gene function, while *‘pred’* indicates predicted gene function according to the gene-specific FlyBase report.

GO TERM	name of affected gene (log_2_FC)	fold-enrichment
*GO:0006739*∼NADP metabolic process	FBgn0004057 // CG12529 (−1.42)	81.9
	glucose-6-phosphate dehydrogenase *^rep^*	
	FBgn0023477 // CG2827 (−1.27)	
	transaldolase *^rep^*	
	FBgn0004654 // CG3724 (−2.15)	
	phosphogluconate dehydrogenase *^rep^*	
	FBgn0030239 // CG17333 (−1.34)	
	6-phosphogluconolactonase *^pred^*	
	FBgn0037607 // CG8036 (−1.39)	
	transketolase *^pred^*	
*GO:0006769*∼nicotinamide metabolic process	same five genes	64.4
*GO:0019362*∼pyridine nucleotide metabolic process	same five genes	60.1
*GO:0006006*∼glucose metabolic process	same five genes, and	16.4
	FBgn0002569 // CG8694 (−0.89)	
	maltase A2 *^rep^*

Lipid storage droplet-2 protein (Lsd-2), a protector against lipolysis and the *Drosophila* perilipin homologue, is localized on the outer membrane of lipid droplets, the main storage organelle for TAGs [44,45]. We found that the level of Lsd-2 in *poly* larval extracts was decreased relative to wild-type fat body, consistent with the decreased TAG : protein ratio observed in *poly* mutants (figure 6*b*).

Together, the reduction in TAG storage and Lsd-2 protein in *poly* mutant larvae suggest a decrease in anabolic metabolism, consistent with—and an expected consequence of—diminished InR–TOR signalling.

### 3.9. The Poly protein relocalizes following insulin stimulation in *Drosophila* haemocytes and human cultured cells

Given the physical interaction of Poly with the InR and functional links between Poly and InR–TOR signalling, we were interested to determine whether the level and/or distribution of Poly in the cell were dependent on InR activity.

Strikingly, staining for Poly was noticeably increased following insulin stimulation of larval haemocytes (cells of the innate immune system). Prior to isolation of haemocytes, larvae were starved for 3 h in 20 per cent sucrose. Haemocytes were then stimulated in culture with 200 nM insulin (electronic supplementary  material, figure S3). This increased staining in Poly appears to be a rapid response as it is detectable following only 15 min of insulin stimulation, and levels of Poly remain elevated after 75 min.

**Figure 7.**
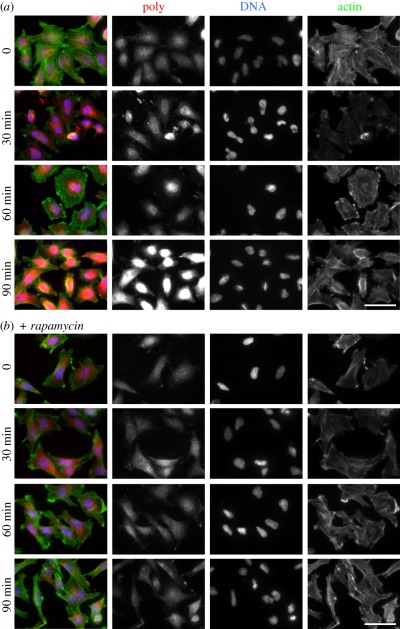
Insulin stimulation of HeLa cells leads to an increase in HsPoly staining, accumulated in the nuclear area. (*a*) HeLa cells were serum-starved overnight and then stimulated with 100 nM insulin for 30, 60 and 90 min prior to staining for HsPoly (red), DNA (blue) and F-actin (green). Staining for HsPoly indicated peri-nuclear accumulation already by 30 min, reaching a maximum following 90 min of insulin stimulation. (*b*) The relocalization of HsPoly was inhibited by overnight incubation of cells with 20 nM rapamycin prior to insulin treatment. Scale bars, 50 µm.

A change in the appearance of Poly following insulin stimulation was conserved in human cells. Overnight serum-starved HeLa cells were stimulated with insulin for up to 90 min. As in *Drosophila* haemocytes, staining for HsPoly was significantly stronger following insulin stimulation, appearing maximal at 90 min. It additionally appeared that this increase in HsPoly was concentrated in or near the nucleus, with relocalization already evident by 30 min of insulin stimulation (figure 7*a*). Nearly, 60 per cent of cells showed a relocalization of Poly by 60 min of insulin stimulation, with this level increasing to 100 per cent by 90 min.

This rapid change in HsPoly behaviour was dependent on TOR signalling. Incubation of HeLa cells with rapamycin during the overnight starvation period prevented the increase in HsPoly staining following insulin stimulation (figure 7*b*). In rapamycin-treated cells, HsPoly remained evenly distributed throughout the cell even following 90 min of insulin treatment.

Taken together, these observations demonstrate that the increase and nuclear relocalization of HsPoly following insulin treatment occurs in a TOR-dependent manner. Overall, our results identify Poly as a novel component of InR–TOR signalling that is conserved from flies to humans.

## 4. Discussion

Poly is a conserved protein that plays a novel essential role in InR signalling and, crucially, promotes the effects of the TOR kinase on cell growth and metabolism.

### 4.1. The activity of InR signalling is decreased in poly larvae

Our data reveal an essential involvement for Poly in InR signalling, an important pathway linking nutritional status to metabolism and cell growth [3]. The two kinases, Akt and S6K, are key effectors of InR–TOR signalling. The Akt kinase is located upstream of the TSC1–TSC2 complex in this pathway and has a critical role in promoting cell growth. Phosphorylation of Akt substrates contributes to a range of cellular processes including cell growth, proliferation and survival [46]. Dysregulation of Akt is involved in various diseases, including type-2 diabetes and cancer [47,48]. S6K kinase is one of the most downstream effectors of InR–TOR signalling, and is subject to phosphorylation and activation by TOR. Activation of S6K leads to an increase in translation through its phosphorylation and activation of ribosomal protein S6 [1].

Poly acts upstream of both of these kinases in the InR signalling pathway, as levels of phosphorylated (active) dAkt and dS6K kinases were decreased in *poly* larvae. That Poly acts in the activation of both dAkt and dS6K is also supported by genetic data that revealed suppression of a *poly*-induced rough eye phenotype by mutations in *dAkt* and *dS6K*. These two kinases should only act as genetic suppressors if they are situated downstream of *poly* in the signalling pathway, resulting in an overall decrease in InR–TOR signalling in the absence of Poly.

The biochemical data presented herein revealed a physical interaction of Poly with InR. Intriguingly, the level of Poly was reduced in *InR* mutant larval extracts independently of transcription, suggesting that a fraction of Poly may be unstable and subject to degradation in the absence of InR. Indeed, there are numerous examples of such instability if one partner in a protein complex is absent [49–51]. Whether Poly exists in a discrete complex with InR, and how such complex(es) may be regulated in response to insulin stimulation, are currently under investigation. Such an interaction may well be transient and highly dynamic, as Poly appears to accumulate in the nucleus following insulin stimulation. Considering the insulin-induced, TOR-dependent nuclear enrichment of Poly in HeLa cells, it is likely that insulin-mediated signalling via Poly is conserved from flies to humans.

Together our data predict a decrease in InR–TOR signalling in the absence of Poly. As one read-out of TOR signalling, we examined autophagy, a multi-step, catabolic process used for nutrient recycling during development and starvation. TOR activity is responsible for inhibiting autophagy under cell growth-promoting conditions. Our finding that autophagy is constitutively active in the fat body of *poly* mutant larvae is in accord with the observation of reduced InR–TOR signalling, and consistent with an inhibition of cell growth and/or proliferation in *poly* mutant animals. Furthermore, the increase in apoptotic cell death in *poly* mutant animals and clones generated in imaginal discs might result from increased levels of autophagy upon mutation of *poly*.

### 4.2. Metabolism is disrupted in poly mutants

A major function of the InR–TOR pathway is the modulation of metabolism. For example, *4E-BP* mutant animals show increased sensitivity to starvation [16]. Interestingly, it was previously shown that the loss of the tumour suppressor *PTEN* (responsible for the dephosphorylation of PIP_3_) in *Drosophila* nurse cells results in the accumulation of activated Akt in the cytoplasm. This activated Akt drives the formation of enlarged lipid droplets along with an increase in the expression of *Drosophila* Lsd-2 [52]. *Lsd-2* mutants are characterized by decreased TAG levels [45]. Interestingly, autophagy and lipid metabolism were shown to be two interlinked processes, as suggested by a decrease in autophagy resulting in an increase of lipid storage in the cell [53]. Consistent with decreased InR–TOR signalling in the absence of *poly*, both Lsd-2 and the TAG : protein ratio were reduced in *poly* mutant animals. Therefore, we propose that Poly affects metabolism via its interaction with the InR–TOR pathway, acting as a positive regulator of anabolic metabolism.

### 4.3. Comparison of poly to other mutants and Elongator

Numerous aspects of the *poly* phenotype have been observed in other mutations, including that of *InR* and *TOR*. An extreme larval longevity, one of the most remarkable aspects of the *poly* mutant phenotype, has been observed in both *Tor* [54] and *InR* mutants (this study). The appearance of melanotic masses is frequently seen in mutations with aberrant immune responses and/or haematopoietic defects [55–57]. In *poly* third instar larvae, melanotic masses appearing at multiple different locations along the larval body are likely to result from either an alteration in the immune response and/or a hyper-proliferation of blood cells, or haemocytes. The observation that the few *InR* mutant larvae that reach the third instar stage also develop melanotic masses highlights the phenotypic similarities of *poly* and *InR* loss-of-function mutations, and further corroborates a functional relationship between *poly* and *InR*.

Given the apparent similarity of Poly to yeast Elp6, it is therefore intriguing that the recently described mutant phenotype for the Elongator Elp3 catalytic subunit in *Drosophila* resembles that of *poly* [28]. These observations are suggestive of a role for the Elongator holocomplex in insulin signalling—through histone and/or tubulin acetylation—or even translational control through tRNA modification. In addition, as human mutations in Elp3 have been linked to familial dysautonomia [58], it is likely that the future analysis of Elongator function in model organisms will be of significant preclinical value.

### 4.4. Model for Poly action

We have shown that Poly binds to InR (a transmembrane receptor) and modulates the activity of various downstream proteins. While the lack of clearly discernible functional motifs hampered a prediction of Poly's molecular function, extensive database searches and phylogenetic analyses identified Poly as a member of the Elp6 subfamily of Elongator proteins, with more distant homology to various members of the RecA/Rad51/DCM1 superfamily (such as KaiC and RadB); these observations are described in a distinct study from our laboratory. Both KaiC and Elp6 have been shown to significantly affect gene expression, pointing to a degree of functional conservation. Thus, we suggest an involvement for Poly during transcription (perhaps once relocated to the nucleus following insulin stimulation).

If Poly has a role during transcription, as the phylogenetic data suggest, how do we explain its binding to a transmembrane receptor? One explanation is the dynamic changes in the cellular localization of Poly, occurring in a TOR-dependent manner. Interestingly, InR phosphorylation of IRS-1 and IRS-2 (two of the human IRS homologues) not only leads to activation of PI3K signalling, but is also associated with IRS-1 translocation to the nucleus, where it activates transcription of various genes [59–61]. Given that Poly also interacts with InR, and moves to the nucleus following insulin stimulation, it is possible that changes in the localization of Poly are also ultimately in response to InR signalling. Immunofluorescence on both *Drosophila* haemocytes and HeLa cells demonstrated that the level and/or distribution of Poly are significantly affected following insulin stimulation. Importantly, in HeLa cells, Poly relocalization to the nucleus occurred in a rapamycin-sensitive manner. Thus, we speculate that the stimulatory effects of Poly on cell growth and metabolism may be exerted via effects on transcription. However, further analysis is required to assess whether Poly participates in an Elongator complex and, if so, in which of the myriad functions currently ascribed to Elongator.

Future research will address the detailed nature of the interaction of Poly with the InR. Does Poly interact with the InR in the absence or presence of insulin? How dynamic is this interaction? And is the level or post-translational state of Poly modified upon insulin treatment?

**Figure 8.**
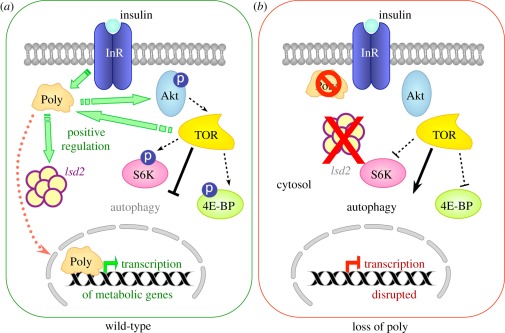
Model for the function of Poly in the InR–TOR signalling pathway. The interaction of Poly with (*a*) the insulin receptor allows it to fulfil a role as a positive regulator of InR–TOR signalling, which has the overall effect of increasing the phosphorylation and, hence, activity of numerous positive regulators of the InR–TOR pathway, such as Akt, S6K and 4E-BP, as well as inhibiting negative regulators of cell growth such as autophagy. Furthermore, Poly aids to promote anabolic metabolism resulting in an increase of TAG and Lsd-2 levels. (*b*) The absence of Poly leads to a decrease in the activation of positive regulators of cell growth such as Akt and S6K kinases. The 4E-BP protein becomes hypo-phosphorylated, consistent with an overall downregulation of InR–TOR signalling. Furthermore, autophagy—a negative effector of InR/TOR signalling—becomes constitutively active. Anabolic metabolism is downregulated as indicated by a decrease in TAG and Lsd-2 levels.

In the light of the data presented herein, we propose that Poly is a novel mediator of InR–TOR signalling in the regulation of cell metabolism and growth in *Drosophila* (figure 8). We suggest that the physical interaction between Poly and the InR is followed by the translocation of Poly into the nucleus (upon insulin stimulation), wherein the expression of key metabolic genes is affected, thus contributing to the promotion of cell growth and metabolism. While the detailed nature and regulation of Poly's interaction with the InR remain to be elucidated, it is highly likely that, given the conservation of Poly, this crucial interaction and function will also hold true in human cells.

## 5. Material and methods

### 5.1. Drosophila culture

All fly stocks were maintained at 25°C on standard medium unless otherwise stated. Fly stocks used in this study were: Canton S (wild-type), *poly^05137^*/TM6B. The following fly lines were obtained from the Bloomington Stock Center: *InR^05545^*/TM3, *Akt1^04226^*/TM3, *S6K^07084^*/TM3, Cg-Gal4.

### 5.2. Phylogenetic analysis

Sequences homologous to *Drosophila* Poly were identified by multiple iterative searches using the PSI-BLAST program [62] and the HHpred interactive server [63]. Alignments between the corresponding sequences were generated using M-Coffee [64] and manually adjusted based on predicted secondary structure according to Ali2D [65]. Identical and similar amino acids in a representative subset of aligned sequences were shaded using the BOXSHADE server (http://www.ch.embnet.org/software/BOX_form.html). Phylogenetic trees were calculated using TREE-PUZZLE [66], MrBayes [67] and the Proml program of the PHYLIP Package [68] after clustering of related sequences into smaller groups using SplitsTree4 [69]. Branch lengths were calculated by application of the WAG substitution matrix [70] using TREE-PUZZLE, modelling rate heterogeneity according to a gamma distribution with 16 rate categories, and bootstrap confidence intervals (provided in electronic supplementary material, figure S1) were estimated using the Seqboot program of the PHYLIP Package [68].

### 5.3. Immunoprecipitation

Fifty microlitres of 0–5 h wild-type embryos (raised at 18°C) were homogenized in 1 ml of cold lysis buffer (100 mM NaCl, 10 mM EDTA, 50 mM Tris–HCl pH 7.6, 0.1% Triton-X100, 10 µg ml^−1^ each of chymostatin, leupeptin, antipain and pepstatin, and 50 µg ml^−1^ PMSF) and briefly sonicated. Eight microlitres of rabbit pre-immune or anti-poly serum coupled to 40 µl of Dynal beads (Invitrogen) were added to pre-cleared embryo lysates overnight. Ten per cent of each sample was subjected to immunoblot analysis to verify successful immunoprecipitation of Poly, while the remaining 90 per cent of sample was resolved on precast 4–12 per cent Bis-Tris polyacrylamide gels (Novex) and stained with Colloidal Coomassie Blue (Invitrogen). Comparable molecular weight regions of interest were excised from each lane (pre-immune- and immune-precipitations) and mass spectrometry analysis performed (Dr Gerard Cagney, Dublin).

### 5.4. Mass spectrometry

The proteins in slices from sodium dodecyl sulphate polyacrylamide gel electrophoresis (SDS–PAGE) gels were digested in-gel with trypsin by the method of Shevchenko *et al*. [71]. The resulting peptide mixtures were resuspended in 1 per cent formic acid and analysed by nano-electrospray liquid chromatography mass spectrometry (nano-LC MS/MS). A high-performance liquid chromatography (HPLC) instrument (Dionex, UK) was interfaced with an LTQ ion trap mass spectrometer (ThermoFinnigan, CA). Chromatography buffer solutions (buffer A: 5% acetonitrile and 0.1% formic acid; buffer B: 80% acetonitrile and 0.1% formic acid) were used to deliver a 60 min gradient (35 min to 45% buffer B, 10 min to 90%, hold 10 min, 3 min to 5% and hold for 15 min). A flow rate of 2 µl min^−1^ was used at the electrospray source. One full scan was followed by 10 MS/MS events, and the duty cycle programmed to enforce dynamic exclusion for 2 min. In-house proteomics pipeline software (Proline) was used to process data. Spectra were searched using the Sequest algorithm [72] against SwissProt.2007.04.19 database restricted to *Drosophila melanogaster* entries. Proteins with (i) peptide prophet probability score greater than 0.99 [73] and (ii) identified by a minimum of two different peptide spectra were automatically accepted, while spectra for the minority of proteins identified by single spectra were manually checked for quality.

### 5.5. Larval manipulations

Prior to dissections or protein extractions, 10–15 second instar larvae were transferred to a fresh vial of food supplemented with fresh yeast paste. Manipulations were carried out when larvae reached early third instar stage. For starvation experiments, early third instar larvae were starved in 20 per cent sucrose solution for 3–4 h prior to dissection.

### 5.6. RNA extraction and quantitative reverse transcriptase–polymerase chain reaction

Larvae were transferred to RNase-free Eppendorf tubes (Ambion). RNA was extracted with the Qiagen RNeasy Plus Kit (Qiagen Hilden, Germany) according to manufacturer's instructions. cDNA synthesis was carried out by using the SuperScript III (Invitrogen) following the manufacturer's instructions. Real-time quantitative RT-PCR analysis was performed on the LightCycler system (Roche) using the universal probe library. The reactions were set up following manufacturer's recommendation with the LightCycler master mix kit. The relative cDNA ratio was calculated with Lightcycler software 480. Actin5C was used as control to normalize equal loading of template cDNA.

### 5.7. Preparation of larval protein extracts for immunoblotting

Fifteen to twenty wandering third instar larvae of the appropriate genotype were placed in 1.5 ml tubes and rinsed three times with Ephrussi–Beadle Ringer's solution (130 mM NaCl, 4.7 mM KCl, 1.9 mM CaCl_2_, 10 mM HEPES and pH 6.9). Next, 300µl of cold lysis buffer (Ephrussi–Beadle Ringer's solution with 10 mM EDTA, 10 mM DTT, 1 µg ml^−1^ of each of chymostatin, leupeptin, antipain and pepstatin [CLAP, Sigma], 1 mM phenylmethanesulphonyl fluoride [PMSF, Sigma], and 1 unit of Aprotinin [Calbiochem] was added to the tubes. The larvae were then homogenized using a motorized hand pestle starting at lowest speed and gently increasing the speed to the maximum for approximately 2 min. Then, 150 µl of hot (70°C) 3X SDS-PAGE Sample Buffer containing 10 mM DTT was added to the homogenate and the tube placed at 100°C for 10 min. Particulate matter was pelleted at 13 000 r.p.m. for 2 min, and the supernatant transferred to a fresh tube. Samples were stored at −20°C until required.

### 5.8. Sodium dodecyl sulphate polyacrylamide gel electrophoresis and immunoblotting

Protein samples were resolved at 170 V by SDS-PAGE on precast 4–12% Bis-Tris polyacrylamide gels (Novex) and transferred onto nitrocellulose membranes in a Trans-Blot apparatus (Biorad). Membranes were blocked in TBSTw (Tris-buffered saline (150 mM NaCl, 20 mM Tris pH 7.5) + 0.05% Tween 20) and 5% (w/v) semi-skimmed milk for 1 h at room temperature and then incubated for 1 h with primary antibody in TBSTw. After washing three times for 5 min with TBSTw, the membranes were incubated in an appropriate horseradish peroxidase-linked secondary antibody (1 : 10 000) for 1 h in TBSTw at room temperature. Finally, the membranes were washed as above in Tris-buffered saline plus 0.2% Triton X and immune-complexes detected by enhanced chemiluminescence (ECL; Amersham Biosciences).

Primary antibodies and dilutions used in immunoblotting experiments were as follows: DmPoly 504 (1 : 1000), anti-Isd2 (1 : 2000), p-Akt antibody (1 : 1000), p-S6K antibody (1 : 1000) and p-4E-BP antibody (1 : 200). The anti-Isd2 antibody was a generous gift from Ronald Kühnlein (MPI, Göttingen), while the last three were purchased from Cell Signaling Technology.

### 5.9. Lysotracker staining

Lysotracker staining was performed as described [74]. Fat body from fed or starved larvae was dissected in PBS, and incubated for 2 min in 100 nM LysoTracker Red DND-99 (Molecular Probes), with 1 µM Hoechst 33342 (Molecular Probes) in PBS. Fat bodies were mounted with phosphate-buffered saline (PBS) on a glass slide and visualized using an Olympus AX-70 Provis epifluorescence microscope and Hamamatsu Orca II charge coupled device (CCD) camera. Images were captured using SmartCapture 3 and processed using Adobe Photoshop.

### 5.10. Microarray processing and analysis

Ten to fifteen second instar wild-type Canton S and *poly* larvae were transferred into vials supplemented with fresh yeast paste. Early third instar larvae were transferred into TRIzol 24 h later for RNA extraction. All microarray processing was by the Flychip team at the University of Cambridge (http://www.flychip.org.uk/). RNA from wild-type Canton S and *poly* early third instar larvae was extracted (medium scale) using TRIzol (http://www.flychip.org.uk/protocols/gene_expression/standard_extraction.php). RNA was reverse transcribed and labelled using Klenow labelling: 5 µg of total RNA were reverse transcribed to cDNA (anchored oligo (dT)23 (Sigma), Superscript III (Invitrogen)) and second strand synthesis was then performed (Second strand buffer (Invitrogen), DNA Polymerase I (Invitrogen), RNaseH (New England Biolabs), *Escherichia coli* DNA ligase (GE Healthcare)) to obtain double-stranded DNA. Random primers are annealed to 500 ng of this denatured DNA template and extended by Klenow fragment using the Bioprime DNA Labeling System (Invitrogen), while fluorescent dyes Cy3-dCTP or Cy5-dCTP (GE Healthcare) are incorporated (http://www.flychip.org.uk/protocols/gene_expression/klenow_v2.php). Hybridization to FL002 microarrays: hybridization to amino-modified long oligonucleotide microarrays using a Genomic Solutions hybridization station with the Biosolutions hybridization buffer (http://www.flychip.org.uk/protocols/gene_expression/hyb_oligoMWG.php). Scanning was with the Genepix 4000B dual laser scanner at 5 µM pixel resolution (http://www.flychip.org.uk/protocols/gene_expression/scanning2.php). Spot finding and quantitation were via the Dapple package. Raw data were normalized by the vsn method in bioconductor (http://www.bioconductor.org/packages/2.8/bioc/html/vsn.html) to generate log (base 2) fold changes and average signals. Differential expression was tested using Limma, also in bioconductor (http://www.bioconductor.org/packages/2.8/bioc/html/limma.html). GO and KEGG biological pathway enrichment in the differentially expressed gene set were assessed using the DAVID functional annotation bioinformatics microarray analysis tool (http://david.abcc.ncifcrf.gov/). Microarray data were deposited in the gene expression omnibus (GEO) under the accession number GSE32637 (http://www.ncbi.nlm.nih.gov/geo/query/acc.cgi?acc=GSE32637).

### 5.11. Triglyceride assay on larvae

TAG assay on larvae was carried out as described by Gronke *et al*. [75]. Briefly, six whole larvae corresponding to each genotype were collected in 500 µl of 0.05 per cent Tween 20 and homogenized using a Polytron apparatus, followed by treatment at 70°C for 5 min. Samples were centrifuged for 1 min at 3500 r.p.m. and 350 µl of the supernatant were transferred to a new vial and centrifuged for 3 min at 2500 r.p.m. Six hundred microlitres of Thermo Infinity Trig solution (Thermo Electron, 981786) were added to 75 µl of isolated supernatant and the absorbance at 540 nm was measured following incubation for 30 min. Similarly, protein content was determined by adding 10 µl of isolated supernatant to Bradford reagent (Sigma, B6916) and reading the absorbance at 595 nm. TAG levels were normalized to corresponding protein levels.

### 5.12. Antibody staining of haemocytes

Third instar larvae were bled on multispot microscope slides (Hendley-Essex) using a pair of forceps and a 25-gauge needle in 20 μl of PBS. Cells were left to settle on the slide for 1 h at room temperature in a humidified chamber to allow adherence to the slide. Cells were fixed with 20 µl of 3.7 per cent paraformaldehyde in PBS for 5 min. Cells were washed with PBS for 5 min, followed by a 5 min permeabilization in PBS + 0.1% Triton X-100. Following an additional 5 min wash in PBS, blocking was performed by incubating cells in PBS + 3% BSA for 1 h. Cells were then incubated with primary antibody diluted in PBS + 3% BSA overnight at 4°C. The following day cells were washed in PBS three times and incubated at room temperature with secondary antibody diluted in PBS + 3% BSA. Following the secondary antibody, incubation cells were washed twice in PBS for 5 min. Cells were incubated with DAPI diluted in PBS (1 : 5000 dilution) for 5 min followed by a final 5 min wash in PBS. Coverslips were mounted with mowiol on top of the slide.

Primary rabbit anti-DmPoly serum was used in immunofluorescence experiments at a 1 : 1000 dilution. Alexa Fluor-488 anti-rabbit (1 : 500) and Alexa Fluor-594 conjugated phalloidin (1 : 500) were purchased from Molecular Probes.

### 5.13. HeLa cell culture

HeLa cells were maintained in Dulbecco's modified Eagle's medium (DMEM; Sigma) supplemented with 10 per cent foetal bovine serum (FBS; Gibco). Cells were grown to 80–90% confluence before overnight serum withdrawal. For insulin stimulation, serum-starved cells were treated with 100 nM insulin (Sigma). Cells were treated with 20 nM rapamycin (Cell Signaling Technology) during an overnight serum starvation.

### 5.14. Immunofluorescence on HeLa cells

HeLa cells were cultured on coverslips in six-well plates overnight. Cells were rinsed in PBS for 3 min followed by a 3 min fixation in PBS containing 4 per cent paraformaldehyde (diluted from 16% ampoules). The fixative was removed by a 2 min wash in PBS. Cells were permeabilized by a 5 min incubation in PBS + 0.5% Triton X-100 followed by a 1 h block in PBS containing 3 per cent BSA. Subsequently, cells were washed in PBS + 0.1% Triton X-100 for 5 min. Primary antibody was diluted in PBS + 0.3% BSA + 0.1% Triton X-100 and incubated on the cells for 1 h at room temperature. Cells were washed twice for 5 min followed by a 1 h secondary antibody incubation diluted in PBS + 0.3% BSA + 0.1% Triton X-100. Subsequently, 45 min washes were performed in PBS + 0.1% Triton X-100. DAPI was included in the penultimate wash at 0.1 µg ml^−1^ concentration.

Primary rabbit anti-HsPoly serum was used in immunofluorescence experiments at a 1 : 1000 dilution. Texas-Red conjugated goat anti-rabbit (1 : 500) secondary antibody and Alexa Fluor-488-conjugated phalloidin (1 : 500) were purchased from Molecular Probes.

## Supplementary Material

Supplemental Figure 1. Phylogenetic tree with bootstrap scores and log likelihood, along with multiple sequence alignment used to generate phylogenetic tree

## Supplementary Material

Supplemental Figure 2. Examination of p-Akt in whole mount larval brains

## Supplementary Material

Supplemental Figure 3. Poly immunostaining increases following insulin stimulation of hemocytes isolated from starved larvae
