# Supplementary material for: Drosophila poly suggests a novel role for the Elongator complex in insulin receptor–target of rapamycin signalling
Source: Open Biol. 2012 Jan;2(1):110031. doi: 10.1098/rsob.110031 (PMC3352090; doi:10.1098/rsob.110031)
Supplement: Supplemental Figure 1. Phylogenetic tree with bootstrap scores and log likelihood, along with multiple sequence alignment used to generate phylogenetic tree [file rsob110031-s1.pdf]

Supplemental Figure 1.

Phylogenetic tree with bootstrap scores and log likelihood

```
[ lh=-21535.300483 ]
((Dm-ELP6:1.48350,((Sp-ELP6:1.74452,((Dd-ELP6:1.21071,(Dr-ELP6:0.48570,
Hs-ELP6:0.28867)100:0.71398)34:0.15259,(At-ELP6:0.39007,Os-ELP6:0.76777)
100:0.76188)59:0.26793)17:0.16713,(Ce-ELP6:2.20689,Sc-ELP6:2.49945)
8:0.21509)5:0.23315)52:0.27081,(((Ce-ELP4:1.45678,(Dm-ELP4:0.68995,
(Hs-ELP4:0.38000,Dr-ELP4:0.25900)100:0.43373)94:0.14141)48:0.17377,
(Sc-ELP4:1.36703,Sp-ELP4:0.90267)81:0.52270)35:0.10395,(Dd-ELP4:0.74628,
(At-ELP4:0.40014,Os-ELP4:0.24954)100:0.92742)65:0.15752)100:2.08676,
(((Dr-ELP5:0.52540,Hs-ELP5:1.21941)96:0.48139,(Ce-ELP5:2.33442,Dm-ELP5:1.92399)
93:0.82266)36:0.35623,(Dd-ELP5:1.25741,((Sc-ELP5:1.18508,Sp-ELP5:1.07219)
99:0.83840,(Os-ELP5:0.62819,At-ELP5:0.47856)100:0.88252)65:0.60569)
26:0.07326)99:1.20760);
```

Multiple sequence alignment used to generate phylogenetic tree

```
>Hs-ELP6 gi|89145415|ref|NP_001026873.2| transmembrane protein 103 [Homo
sapiens]
--------------------------------------------------------------------------------
-------MFVELNNLLNTTPDRAEQ--GKLTLLCDA-K-TDGSFLVHHFLSFYLK--ANC-----------------KVC
FVALIQSFSHYSIVGQKLGV--SLTMAR-------------------------ERGQLVFLEGLKSAVDVVFQAQK---E
PH-P-LQFLREAN-------------------------------------------------------------------
-----------------------------------------AGNLKPLFEFVREALKPVDSGEAR----WT---YPVLLV
DDLSVLLSL-GMG-------AVAVLDFIHYCRATVCW-------------------------------------------
---------ELKGNMVVLVHD-SGDAE--------DEENDIL-LN-GLSHQSHLILRAEGLATG------------FCRD
V-----------------HGQLRILWRRPSQP----AVH--RDQSFTYQYKIQ-DKSVSFFAKGMSPAVL----------
--------------------------------------------------------------------------------
---------------------

>Dr-ELP6 gi|156739327|ref|NP_001096612.1| transmembrane protein 103 [Danio
rerio]
--------------------------------------------------------------------------------
-------MFPELNSLLNASPDTFKP--GDFILLSDR-Q-ADASFLIHHYLSFYLR--AGC-----------------KVC
FLGLVQSFSHYSAVGQRLGV--SLTQAR-------------------------EKGQLVFLEGLKDSIGAILKEDTS--E
GTQA-LSYLRSPR-------------------------------------------------------------------
-----------------------------------------AG-LEGLFRFVESSLCQSGDDG-----------PPVLII
DDLSVLLSL-GVS-------AGAILDFTLYCRATVCS-------------------------------------------
---------ELQGNMVILVRC-EEEDAD-----DDEEGLNLL-QR-GLVHQCHLALHVEGLPTG------------YCRD
I-----------------HGQMEVWWRQGEND----AYN----QRKIFQFKVH-DKGASFFTRGTSRAVL----------
--------------------------------------------------------------------------------
---------------------

>Dm-ELP6 gi|7211777|gb|AAF40432.1| DID [Drosophila melanogaster]
--------------------------------------------------------------------------------
-------MATSVLLACGLN-EQKLP--G-FVHISEESN-VDGSFLISCVLGQRLRI-SNA-----------------GTL
LVCLQHHYQHYFNAGMRLGY--NTNIFQ-------------------------GK-TLGVIDVLSDMAGE----------
GL-A-SKWLTNTE-------------------------------------------------------------------
----------------------------------------GQTLTEQLVEDIRAQVESNYANRN----------SYTVLI
DNLSILFNL-GAS-------KLQVQQFCQDLAALGKE-------------------------------------------
---------REKLTVITKLSN-SDIY-------------QLT-DN-NVAKLGQVRIQVLRLKSG------------VFRE
V-----------------DGKLLIERVLDEGN----YAC--EETRKEVLYKVN-DRNVKVFAPGEIGVKV----------
--------------------------------------------------------------------------------
---------------------
```

```
>Ce-ELP6 gi|17560122|ref|NP_504497.1| hypothetical protein F25B4.4
[Caenorhabditis elegans]
--------------------------------------------------------------------------------
--------MLKIL-K--GQ-EESIK--G-LIVCEEVDN-ASSLPFVLHFLSTAST--SSQ-----------------KVA
IVSTKLSETNYKLICSKAGV--RWNPTQ-----------------------------ISFYDFLKP-FELF---------
--------------------------------------------------------------------------------
-----------------------------------------DITSEQIMDELYKKLIDSS--------------ASVILF
DDLSILEQFGATS--------VETTIFVHKIYTHLKT-------------------------------------------
---------LSQENALLLASF-SIHS--------------NA-VD-ILKPRCHVFVQMSPVSHG------------FGKD
A-----------------SAKAVLTIKSPSAP-----------TSTKGILLSG-ERTINGSYIT---VDQ----------
--------------------------------------------------------------------------------
---------------------

>Os-ELP6 gi|24796809|gb|AAN64485.1| unknown protein [Oryza sativa (japonica
cultivar-group)]
--------------------------------------------------------------------------------
--------MEEYGGGDLLS-EAMGS-GARVVVVEDRVE-APGAFALHLLLKRALA--GGG-----------------AAA
LLALAQPFSHYDRVLRKMIF--NAVELT--------------------------HLLVLIKDGWEKQNSTPSVSQR----
------GAKGGAI-------------------------------------------------------------------
-----------------------------------------SDSFVRLYGDIQRAIDASRTGDN-----TG---RFTLMI
DDVSLLEVAAGGS-------VDDVLDFLHYCVTLTSE-------------------------------------------
----------MNCSLVFLIHE-DIYS---------SEEGVGV-LL-HLRYIADLVIRAAPLSTG------------LAAD
V-----------------HGQLSVVNKCTFREQR---LK--AQRIWNFHFRVK-ENGADFFYPGSR---H----------
--------------------------------------------------------------------------------
---------------------

>At-ELP6 gi|18413296|ref|NP_567351.1| unknown protein [Arabidopsis thaliana]
--------------------------------------------------------------------------------
-MDRSLNLLDLALGFDEQL-AIPSPLNGKVILIEDCVE-TSGSFVLHQLMKRVLS--SNS---------------SDALI
FLAFARPFSHYDRILRKLGC--NLATHK-------------------------SNNRLVFFDMLMVKCSDGD--------
------QME-D-N-------------------------------------------------------------------
-----------------------------------------VSAVAKLFREIQETVRKLQSVT------SG---NITVMV
DDMSLLEIATTGS------NSDHVLDFLHYCHTLSSE-------------------------------------------
----------SNCSLVILNHE-DIYA---------SMERPAF-LL-QMVCLADVVIKAEPLASG------------LAND
V-----------------HGQLTVLNKGISNSGR--GSS--RNKLQNFQFRIK-ENGIDYFYPGCR---S----------
--------------------------------------------------------------------------------
---------------------

>Sc-ELP6 gi|6323972|ref|NP_014043.1| Subunit of Elongator complex, which is
required for modification of wobble nucleosides in tRNA; required for Elongator
structural integrity; Elp6p [Saccharomyces cerevisiae]
-----------------------------------------------------------------------------MGS
VQRQDLVLFSDQSVLPAHF-FQDSNS-HNLFFITHQSC-TQPLWMINALVETHVL--GSPSSLNESSSSMLPSSTRSHAV
LASFIHEQNYFTNSLNKLKI---------------------------------PSNNYNVLDFLSDFIVNN---------
-------IH-NKP-------------------------------------------------------------------
----------------------------------------RDKILSDVLAKFSAAIQNN--PT-----------DTIVII
EQPELLLSL-VSG----LTCSELNNKFITPLLRQCK--------------------------------------------
-----------VLIIVSNSDIFNIDEYD----ASVHSSNLQNFYK-SSFIKSMINLNLNPLKTG------------FAKD
V-----------------TGSLHVCRGGAPIATS--NTSL-HVVENEYLYLNE-KESTKLFYR------------------
--------------------------------------------------------------------------------
---------------------

>Sp-ELP6 gi|19112557|ref|NP_595765.1| elongator homolog [Schizosaccharomyces
pombe]
--------------------------------------------------------------------------------
-----------MSSLHEHL-RPIPEP-FSLTLLLGTRE-TPVTFLFHYYLYHALK--AKE-----------------STC
FLTFSKTLDEHAISMRKWGM--DIKTK----------------------------KNFFIDGFSMLFAPISK--------
---P-SKVQ-APE-------------------------------------------------------------------
----------------------------------------TKNHIKSVFAPVIQCVEENDFEFE----------NSTIII
EDIDILQST-------------HALDSTKIQQAILELR------------------------------------------
```

------KCFSRVIVNVTLGAP--LPQ-------------QKSLGS-SIGHMATRCISCRPLTSG------------SARR
I-----------------TGFLRLSRMPNHFRSGICETP--EDDDKELLYEVT-EAGAKVYSKGQVTLQL----------
----

>Dd-ELP6 gi|74848742|sp|Q9GPT6.1|CC075_DICDI RecName: Full=UPF0405 protein
C3orf75 homolog
---MDL
FSHLNWYSGDFMNEDGEPI-KSIPS--GKLILVSDTLE-SEGSFLIHYFLQSIFKATTSTNSSTNNNNSNNSGGVGGGAC
LLGLNQSLYNYFNVGRKLGY--NLTTEY-------------------------NKGNFTFINGLSTPYKWIIEQRLQQLE
DQGIDEEPQLDSISQGFNPFPTIHLIDNKNITSSSSSSSYSNSNN-----------------------------------
--KNKNNNELKDILYKIYNEFINDHKKRVMNNNNNSKTLFII
DGLNLLESHYSTNPSGS---NMDILNFLQYCHNYIKEN--
---------STTCSMIILYHS-DCDE-------------DSKFFN-LLQYESDLTINITGLKSG------------YSKD
I-----------------DGQLNFIQKDEKNN------T--FTRVNPIHYQAL-DNSIRFFSMGSRIQ------------
----

>Hs-ELP4 gi|91208435|ref|NP_061913.3| elongation protein 4 homolog [Homo
sapiens]
----MAAVATCGSVAASTGSAVATASKSNVTSFQRRGPRASVTNDSGPRLVSIAG-------------------TRPSVR
NGQLLVSTGLPALDQL-LG-G-GLAV-GTVLLIIEEDKYNIYSPLLFKYFLAEGIV--NGH----------------TLL
VASAKEDPANILQELPAPLL--DDKCKKEFDE-----------DVYNHKTPESNIKMKIAWRYQLLPKME----------
IG-P-VSSSRFGHYYDASKRMPQELIEASNWH---------GFFLPEKI-----SSTLKVE--P----------------
--------------------------------------CSLTPGYTKLLQFIQNIIYEEGFDGSNPQ--KKQRNILRIGI
QNLGSPLWGDDICCAENGGNSHSLTKFLYVLRGLLRT---
---------SLSACIITMPTH-LIQN-------------KAI-IA-RVTTLSDVVVGLESFIGSERE-T----NP-LYKD
Y-----------------HGLIHIRQIPRLNN-L--ICD--ESDVKDLAFKLK-RKLFTIERLHLPPDLSDTVSR-----
--SSKMDLAESA---------------KRLGPGCGMMAGGKKHLDF----------------------------------
----

>Dr-ELP4 gi|62955253|ref|NP_001017638.1| elongation protein 4 homolog [Danio
rerio]
----MAAPVRGMAVSSSIG---------NTTSFQKK-TRS--------KLVSIPG-------------------TRPSVQ
NGQLLVSPGVSSLDYV-IG-G-GLAV-GTLLLVEEDRYDSYSRMLLKYFLAEGIV--CGH-----------------ELF
LASARDHPDQIMQELPSPIL--DDVASMKMSE-----------GQSQPNDPDNPDTMKIAWRYQNQPRVQ----------
TA-L-ASSSRFGHYYDASKTMDPELLQAAKYH---------SFYQLQET-----PVTTGL--------------------
--------------------------------------SSLPSPYLALLKSIQTLIQKEGFDGSTPQ--LRGRNVLRVGL
HSLGSVLWGDDVCCKDNSAHCHALSTFLYALRGLLRT---
---------SLSVAMMTVPSH-LIQS-------------RAV-MG-RIIRLSDTAIALESFRGSEKE-T----NP-LYKD
Y-----------------HGLLYVLQIPRLNC-L--TSE--VPDTKDLAFKLK-RKQFTIERLHLPPDLSETVSR-----
--VSKADLAA----------------------GCASTATGNKHLHF----------------------------------
----

>Dm-ELP4 gi|19920758|ref|NP_608932.1| CG6907 [Drosophila melanogaster]
-----------------------------MTSFRKRTVQK-----------PIRG-------------------TRTSPH
TAQVITSSGNPYLDVV-IG-G-GLPM-GSICLIEEDRFMTHAKVLAKYFLAEGVI--SKQ-----------------EIF
LGSLDDIPAEMLRRLPRPLT--DQESMEQSEV-------------QALGDAGAENGLRIAWRYNDLPLVN----------
SE-H--ATAKIGHHFNLMEQMDSMMLYNVKTTLWDDSPKHLDIVIDEEFSKSSSPTTPSLEQQPVEDAPPIPGTETAPQE
KMPAQEEENSANNNNNNNNNSSSVTSSTKTGSQDSPLQVFHNPRYKGLLNDIQQLLRNESFVAG------TKNNLCRVCL
TSLGSPLWYDEHF-------GEDLIKFLTLLMASVRN---
---------CNSVCLITMPMH-LIAKYD-----------ASL-VP-KIRQLVDYAIELESFAGSERE-T----HP-AFKE
Y-----------------SGLLHLHKMSAINT-L--AVH--MPETPDLAFKLR-RKKFIIEKFHLPPELQESSAK-----
--PDNCIS------------------------GLLSNSNATASLDF----------------------------------
----

>Ce-ELP4 gi|17538796|ref|NP_501343.1| ELongator complex Protein Component family
member (elpc-4) [Caenorhabditis elegans]
------------ML--NVG-----------------------------DSVQI---------------------AGCSTK
KRLLETSSGCDSFDTL-IG-G-ALVN-SSIVLIDEYRSRCYGSYLIRSFLAEGLH--HGH-----------------RCF

```
IADPTEDPKEINLI-PSRRT--STDMQNQEIP--------------NSLPAENENSMKIAWRYGNVKQVS----------
SS-L--GPLSNENQYDFTKHVENPNVDV-----------------------------YAEK--V----------------
--------------------------------------FTLKGIYE-TLCQIVKENEAHTKSGGRGG--P-KKNLLRVVL
KNIDMEICEDYKF----------LGRFLCCLRSLARS-------------------------------------------
---------SYMIVYITANSF-RVP--------------KPT-WR-ILESAADTHIQLMPFDENEKK-M----FK-HLGT
A-----------------HGYFHLKSLPRLMS-V--GTH--TPPILDLIFEASSRKGFQIRVMHLPPAFDEPAPG-----
----------------------------------HQNTPSCQNIDF----------------------------------
---------------------

>Os-ELP4 gi|115469124|ref|NP_001058161.1| Os06g0639600 [Oryza sativa (japonica
cultivar-group)]
--------MAAAAGGQTVGR----------SSFSRAAAPHV-------ASSSTAAG------------------VKLGPN
GAA-FVSSGIPDLDRI-LG-G-GFLI-GSVVMIMEDSDAPHHLLLLRSFMAQGVV--HKQ-----------------PLL
FAGPMKEPRLFLGTLPAVAS--SKEDGRQRGMG----------AGTSSDGRTSDEALRIAWQYKKYFGEEKTS-------
HAEHRDNKQEFSNDFDLRKPLERHLLNA-----------------------------QNIE--C----------------
----------------------------------------ASTQEGDTLGVLQDRCSTFLSKLPRKD--GGNAHAGRIAI
QSLCAPQCGYFEK-------DWDMVSFIRSLKAMVRA-------------------------------------------
---------SNAVAVITFPNT-VLS--------------SSF-CK-RWQHLADTLLSIKAIPDEDKE-L----AK-LLTG
Y--------------QDMVGFLHVHKVAQTNS-Q--VPV--ILEASTFSLKLRKRRSLVLERLNQAPVDGSGGPS-----
--LDASGS--------------------------CSSSSQGSQLDF----------------------------------
---------------------

>At-ELP4 gi|66841022|emb|CAI79646.1| elongator component
gi|42564060|ref|NP_566388.2| Paxneb protein-related [Arabidopsis thaliana]
------------MAAPNVRS----------ISSFSRNISVV-------SSPQIP-G------------------LKSGPN
GTA-FISSGIRDLDRI-LG-G-GYPL-GSLVMVMEDPEAPHHMDLLRTFMSQGLV--NNQ-----------------PLL
YASPSKDPKGFLGTLPHPAS--SKEDKPTAPDP------------------DQGESLRIAWQYRKYLENQ----------
KN-A---IDDYSNDFDMRKPLERQFLSG-----------------------------RPID--C----------------
----------------------------------------VSLLDSSDLSIAQDHCATFLSKFPRNS--SNIASIGRIAI
QSFCSPLCEYSEK-------ESDMLSFIRLLKSMLMV-------------------------------------------
---------SNAVAIVTFPPS-LLS--------------PSS-SK-RLQHMADTLLSIKAIPDGDKE-L----EK-LLTG
Y--------------KDINGFLNIHKVARINT-Q--VPV--ILEAKTFSMSLKKRRFLALECLNQAPVDGSSGTS-----
--YGTSGS--------------------------CS--SKSGALDF----------------------------------
---------------------

>Sc-ELP4 gi|6325155|ref|NP_015224.1| Subunit of Elongator complex, which is
required for modification of wobble nucleosides in tRNA; required for Elongator
structural integrity; Elp4p [Saccharomyces cerevisiae]
MSFRKRGEILNDRGSGLRGPLLRGPPRTSSTPLRTGNRRAPGNVPLSDTTARLKKLNIADESKTKMGLDSSHVGVRPSPA
TSQPTTSTGSADLDSI-LG-HMGLPL-GNSVLVEEQSTTEFHSILGKLFAAQGIV--HNRISDS----SADKTRNGDTHV
IVLSLN--QMFAKELPGIYKGSRKQMKKNLISEEESKVTVQNLNETQRSTPSRYKDLKIAWKYKLADEKRLGSPD---RD
DIQQNSEYKDYNHQFDITTRLMPAPIAS-------------------------------EL--T----------------
----------------------------------------FIAPTQPVSTILSQIEQTIKRND---------KKLIRIVI
PSLLHPAMYPPKM-----FESSEIIGLMHGVRSLVKKY------------------------------------------
--------YERVVLFASISID-IIT--------------PPL-LV-LLRNMFDSVINLEPFNQEMTEFL----ER-VYKS
Q------------PGKIQHGLVHILKLPVFTD-R--GEM--RVLKSEWAFKNG-RKKFEIEQWGIPVDDAEGSAA-----
--SEQSHSHSHSDE----------------ISHNIPAKKTKISLDY----------------------------------
---------------------

>Sp-ELP4 gi|19075702|ref|NP_588202.1| RNA polymerase II elongator complex
subunit Elp4 (predicted) [Schizosaccharomyces pombe]
------------------------------MSFKRKAAPQI-------APTNLPTG------------------VRLSSK
DARWITSSGSSSFDYY-LS-G-GIPM-KSLLVIEEDSM-DYASVLLKFFAAEGLK--QDH-----------------VIW
LGPSIG--EMWFRQLPGDSDRPNKNENSAGED----------NHSSPPSKNPQQERMKIAWRYEQVSKTKA---------
PT-LDMIPPGYTHSFDLSKNLIVKSDMK-----------------------------YAVS--P----------------
--------------------------------------FPLETGSNPYAPVIESLTRFLSTLTP--------GTVCRLVL
PSILSPAFYSIRA-----THPQHFIRFIHTLSSLIKCT------------------------------------------
-------TSVHLICMCSVPST-LFSR-D-----------CEQ-IF-WLENLASAVFSLHPFPVKET--V----NG-LVTQ
P-----------------LGLFRIHKLPLALP-F--TNHANSNEAGDLSFTVS-KRRFTIEPWVLPPLDDEQKDT-----
--K---------------------------ISNTNPQKQPVKSLDF----------------------------------
```

```
--------------------

>Dd-ELP4  gi|66815887|ref|XP_641960.1| RNA polymerase II elongator complex
subunit [Dictyostelium discoideum AX4]
---MTTPRVPTTFTRKVAGGVKTTTTGSSLPTNNTSTTTTTTTT----AAKKLPNG------------------CKISIK
NSNLLTSTGLTDLDDI-IG-G-GIPI-GSILMIEEDINSSYYMFLLKYFLAEGVL--QQQ-----------------GVF
FSSLIG--IDPFEILNKLPARITKEEEIEADKNDTTN-NINTNINTNNNNKQPTDELKIAWRYQQYVSNELSKQ---QQQ
QSASTIMNQTFCHSYDFTRKMNVQSMNP-----------------------------ELIH--T----------------
------------------------------LSHDAQSQAEGTSPYRNLFLEIQNLVYKYNKEAAMN---PDQTRVLRLCI
QSFSSPLWSNDE---------EGVIEFLHALKGLLRS-------------------------------------------
---------SVATCVISVPTY-IYS--------------GAF-VK-KIAHLCDTVVSINSFSGLGGE-T----PE-QFAE
Y-----------------LGLFNIRKIARLNT-L--SLS-FHPDMLTFVFKMK-RRKMCIETIHLPPESSRAGDS-----
--KPDNNDSNKNKSDQNNIVSKMKSGSGLLCGGGGGGSSNNNPLDF----------------------------------
--------------------

>Hs-ELP5 gi|44662826|ref|NP_981959.1| dermal papilla-derived protein 6 isoform 3
[Homo sapiens]
-------------------------------------------------------------------------------M
TPSEGARAGTGRELEM-LD-SLLALG-GLVLLRDSVEW-EG-RSLLKALVKKSAL--CGE-----------------QVH
ILGCEVSEEEFREGFDSDIN-----------------------------------NRLVYHDFFRDPLNW---------S
KT-E--E-------------------------------------------------------------------------
----------------------------------------------AFPGGPLGALRAMCKRTD-----PV---PVTIAL
DSLSWLLLRL------------PCTTLCQVLHAVSHQDSCP---------------------------------------
-----GDSSSVGKVSVLGLLH-EELHG------------PGP-VG-ALSSLAQTEVTLGGTMGQ----------------
-------------------ASAHILCRRP-------RQR--PTDQTQWFSILP-DFSLDLQEGP--SVESQPYSDPHIPP
-VDPTTHLTFNLHLSKKEREARDSLILPFQFSSEKQQALLRP--------------------------RPGQATSHIFYE
PDAYDDL-D-QE-DPDDDLDI

>Dr-ELP5 gi|121583681|ref|NP_001073536.1| dermal papilla-derived protein 6
homolog [Danio rerio]
--------------------------------------------------------------------------------
-----------MLLEV-LQ-A-AEAG-GFILIQDSVQC-CG-RGILRCCINAALK--RDE-----------------DVH
VLGFESPETEVCAGLDSSFA-----------------------------------QKLHFHKGFPDPLGW---------R
GK-S-SF-------------------------------------------------------------------------
----------------------------------------------TVQQFTSQHITQLIRDSQ-----PA--KASVLVV
DSLSLVLRHH------------DPVIVCQSLQELRKG-------------------------------------------
----------GVVKTIIGLLH-SDLHL------------QGI-VG-IVCHLASTVISVAPTNNERH---------------
-------------------AVATTTRRTK-------SGK--VMQEEEYFSVSE-DATLSVQSKP--RQHDRVEKEQDSAE
-VDPASNLTFNLRLSEEERRAKEKVALPFVFSQEKKSALL----------------------------RPTPGSGRIMYE
PDANDDF-D-EE-DPDDDLDV

>Dm-ELP5 gi|24655951|ref|NP_647704.1| CG2034 [Drosophila melanogaster]
--------------------------------------------------------------------------------
------------MLSN-LV-V-TKQ--KVVLVIDELNR-ERIAPKFIGSLLHEQGQ-GADTI--------------KALP
TGVSLKHVATFEALIDK---------------------------------------------------------------
--------------------------------------------------------------------------------
-----------------------------------------------YANNNTGS-TTDSNST-----------GFNVIL
PTLADLLCYQ------------TPAFIFGFLNRLRRS-------------------------------------------
---------DNVRRVFLWASP-QHL-Q------------DPH-AD-YILAGCEYLAELVLRLESD---------------
-------------------KLLSLISRKP-------GGG--VSNRRYSCEVSKTQFKVTPLDGGL-PAGASPKQPSPEAE
QTTEPASSTFKIELDEDEVLARNALTLPYER-------------------------------------TSEPSEGNIIYT
PDADDDF-D-EE-DPDEDLCI

>Ce-ELP5 gi|115532614|ref|NP_001040822.1| hypothetical protein W09B6.4
[Caenorhabditis elegans]
--------------------------------------------------------------------------------
------------MSTF-YD-A-PKS--SVAVFQYTDPL-EA-LKILRSALQTDKK----------------------SII
---C---HSAFATWYKEFHV--EWNED-----------------------------------------------------
FV-E----------------------------------------------------------------------------
----------------------------------------------FSDESYKNVEKID---------------REVVIF
```

ADFDVFSMIF------------GISKTLNLIKTLKT--
-----------KKRIFIVTSL-DFP-A------------TPH-LE-HLTNSTFKVKKSANPEIFE----------------
-------------------CQTTVFDKK---------GN--LTIMDQIITMPP-SGTK-PTFKTVKKVENS--------AE
NSI-SAGIS---GISLGSESGKAAMDLPFFVSRQEDGVALRDAA-T----------------------KKIRVGGQIVYE
PDQEDDL-D-DS-DPDDDLNI

>Os-ELP5 gi|115451397|ref|NP_001049299.1| Os03g0201700 [Oryza sativa (japonica cultivar-group)]

-------MAEAAVRCL-RDGRLDGEHAPALAVASNIQCGPLAAGAMLHLAAAVASNAAAGKA------------QARGLV
IVAFDRSPEVYLDFMRRRGL--DPNALN---------------------------RCVRILDCYSDPIGW---------N
QK-I-RSQQQQESGADLCSANKEN--
--VTIFRNVKDLDKLMCSTIDLGRGFAG---EG-KIYFSIAV
DSISSMLRRA------------SVSSISSFLSNLRSH---
----------DQISSIFWLIH-SDLHE------------PKF-SR-AFECLSTMVASLEPAVVDSVYE-EE--IPGNISF
L------------EENYSKAKFYLRLKRR-------NGR--VKHLYEELHVEG-NDVR-FVSAP--SVST-EV-------
SQSLLPKVQFNLELSEKECSDKANVVLPFEHQGKGEPIHIYDGRRSLPEAQQDSNLTASALLDEVKFPKSAAPKGEIHYF
RDSDDEQPDSDE-DPDDDLDI

>At-ELP5 gi|42569125|ref|NP_179432.3| unknown protein [Arabidopsis thaliana]

-------MAESIFRKL-RDGGEEGELAPALTIEETVAS-PFGLDVSGYLLTNLSSSILAGKS------------SSQGLV
LITFSRSPSFYLQLLKQKGI--VVSSSS---------------------------KWIRILDCYTDPLGW---------I
DQ-S-STSFSEGSSL---
--IKLHKCVSDLKKLFSSIIEAGRELVG---TG-KTRFCVAI
DSVNELLRHS------------AMPLVSGLLTDLRSH---
----------AQISSVFWSLN-TDLHQ------------EKV-TN-ALEYISTMKANLEPLCPSSDGQ-RN--ALENLFS
V------------HQDFGKGRFHVRFKLR-------KGR--VRVMSEEYHVDQ-SGIN-FSPIS--SVDT-VI-----AA
TKSLLPKVQFNLQLSEKERVEKEKVVLPFEHQDDGKSNEIYDGRRSLVDGKIETTPLSSMELQT--DVVSSGKGGEIIYF
RDSDDEHPDSDE-DPDDDLDI

>Sc-ELP5 gi|6321981|ref|NP_012057.1| Subunit of Elongator complex, which is required for modification of wobble nucleosides in tRNA; iki1 mutations confer resistance to the K. lactis toxin zymocin; Iki1p [Saccharomyces cerevisiae]

MASSSHNPVILLKRIL-SL-T-ESS--PFILCLDSIAQ-TS-YKLIQEFVHQSK---SKGNE--------------YPIV
YISFET---VNKPSYCT---QFIDATQ---------------

--MDFVHLVKQIISYLPAATA----TQ-AKKHMVII
DSLNYIS-T---------------EYITRFLSEIASP---
-----------HCTMVATY-H-KDIKDENRTVIPDWNNNYPD-KLTLLQFMATTIVDIDVVLTG-TLDTEEVSELLNEFR
I-----------PRGLNNDIFQLRLVNKRK------SGR----SLEYDFIVNS-NTHE-YELLST-TKQEEESSSNGLET
PEMLQGLTTFNLGTSNKQKLAKDQVALPFLEAQ------------------------------------SFGQGGAIVYE
YEKDDDY-D-EE-DPYEDPF-

>Sp-ELP5 gi|19112643|ref|NP_595851.1| RNA polymerase II elongator complex subunit [Schizosaccharomyces pombe]

------MSKFLLNRCI-RD----LS--PLTVLKDNLQQ-TA-KPILNYYAKNAA---SRGI----------------KVL
FISYET-LEKEAPEGID---CFLYATS---------------

--WEKVKSLKELYEHI-SSWR----TQ-GKQHIVMI
DTINPILNTS-------------ISSFTMFFGSVLAL---
----------GSICFLTSF-H-KDVTLEN---YPS--YLPPC-EV-FLDFTSTCTVSLIGMQHLSVEHDAKMRSLPNPLL
EELQDDKIISLLGSNCETAIVLHVEFRKK-------SGR----IIKESCVLKN-GKLEPYTPFEE-TARGPEP------A
DNQIDFNVSFNLNVSEKERKERDKVFLPYFSAQMVGSQHK----------------------------SSFVDEGTIIYH
ADEADDF-D-EEEDADEDLLI

>Dd-ELP5 gi|66818973|ref|XP_643146.1| hypothetical protein [Dictyostelium discoideum AX4]

```
-------------------------------------------------------------------------------M
SSTIVSSIGSNSFTKV-FSSSGSDAVGGLILLEDSLES-SS-QHLLNHLYSLWLPTISKQH---------------RNIW
FLNFSSTIS-EYKQLSK-------KYNN---------------------------CNFIVIDYYSDSFGW---------N
NN-N-NNNSNNNNITENLQQIP----------------------------------------------------------
----------------------------------------MIFKSVKHDSASIIKDIQNVYNCVP----NKFKENPIILI
NSISTLILKS------------GLSDTCNLIRSLTNYNFNKVKQDKEKQEKEQKEQQDNNNNNNKKKIEKINKIETGIEG
SVSNRIKMEKSFSCIFAIL-H-TDLHEYE----------QSV-QK-QLQYISSVSIQVTPLSAKLKYSESLP--------
---------------HPYESTITLITKKR-------SGR--VVRNVEYYYINN-ATGKVEFDSAESLQTKQEE------Q
EPDPTQNLSFNLKLTEDEKQARDSVVLPYRHQGN-------------------------------------NNEQTLLIE
DPDDEDF-D-DE-DPDDDLDI
```
