# Supplementary figures and images for: Drosophila poly suggests a novel role for the Elongator complex in insulin receptor–target of rapamycin signalling
Source: Open Biol. 2012 Jan;2(1):110031. doi: 10.1098/rsob.110031 (PMC3352090; doi:10.1098/rsob.110031)

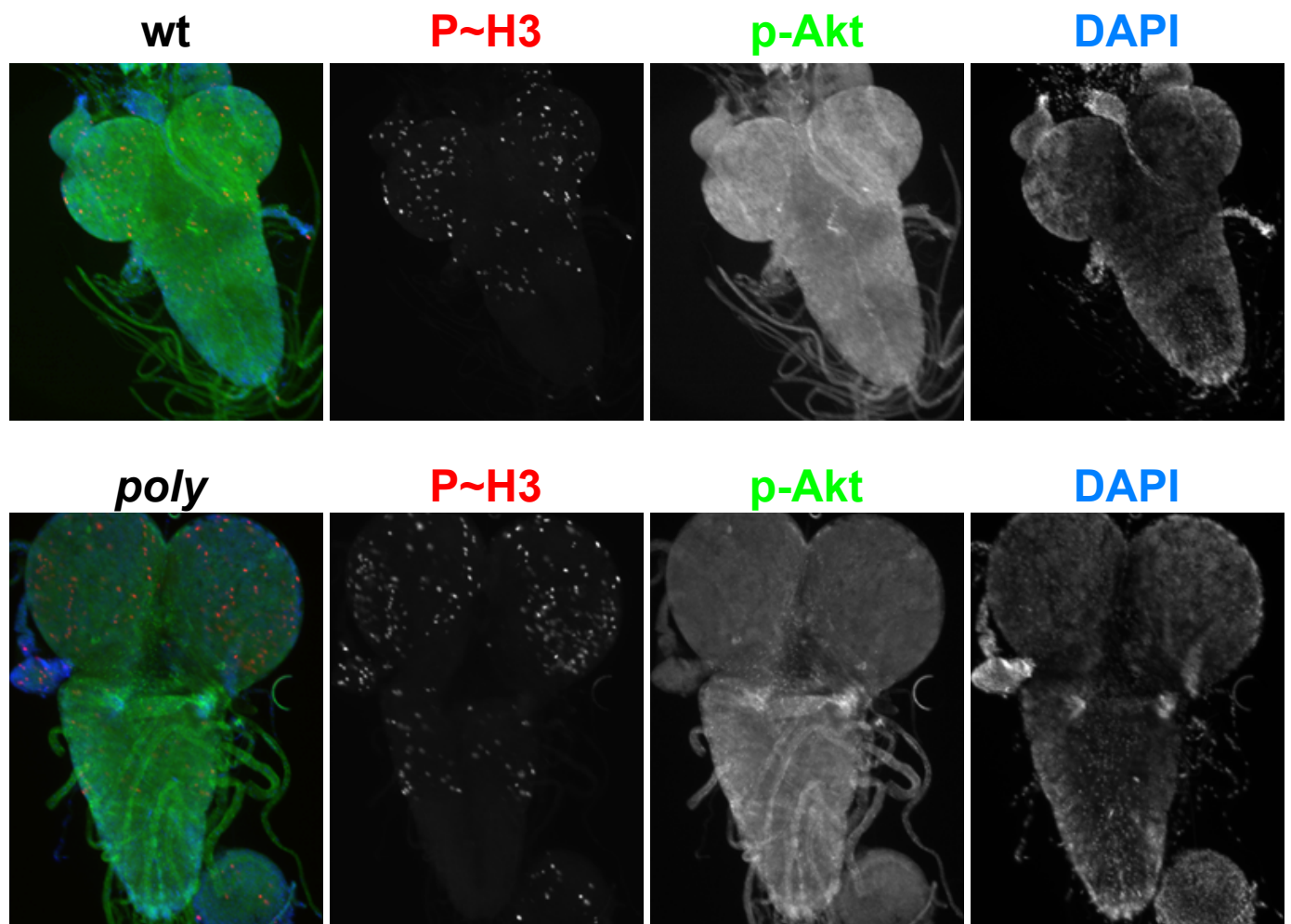

Supplemental Figure 2  
Bolukbasi, *et al.*

Supplement: Supplemental Figure 2. Examination of p-Akt in whole mount larval brains [file rsob110031-s2.pdf]

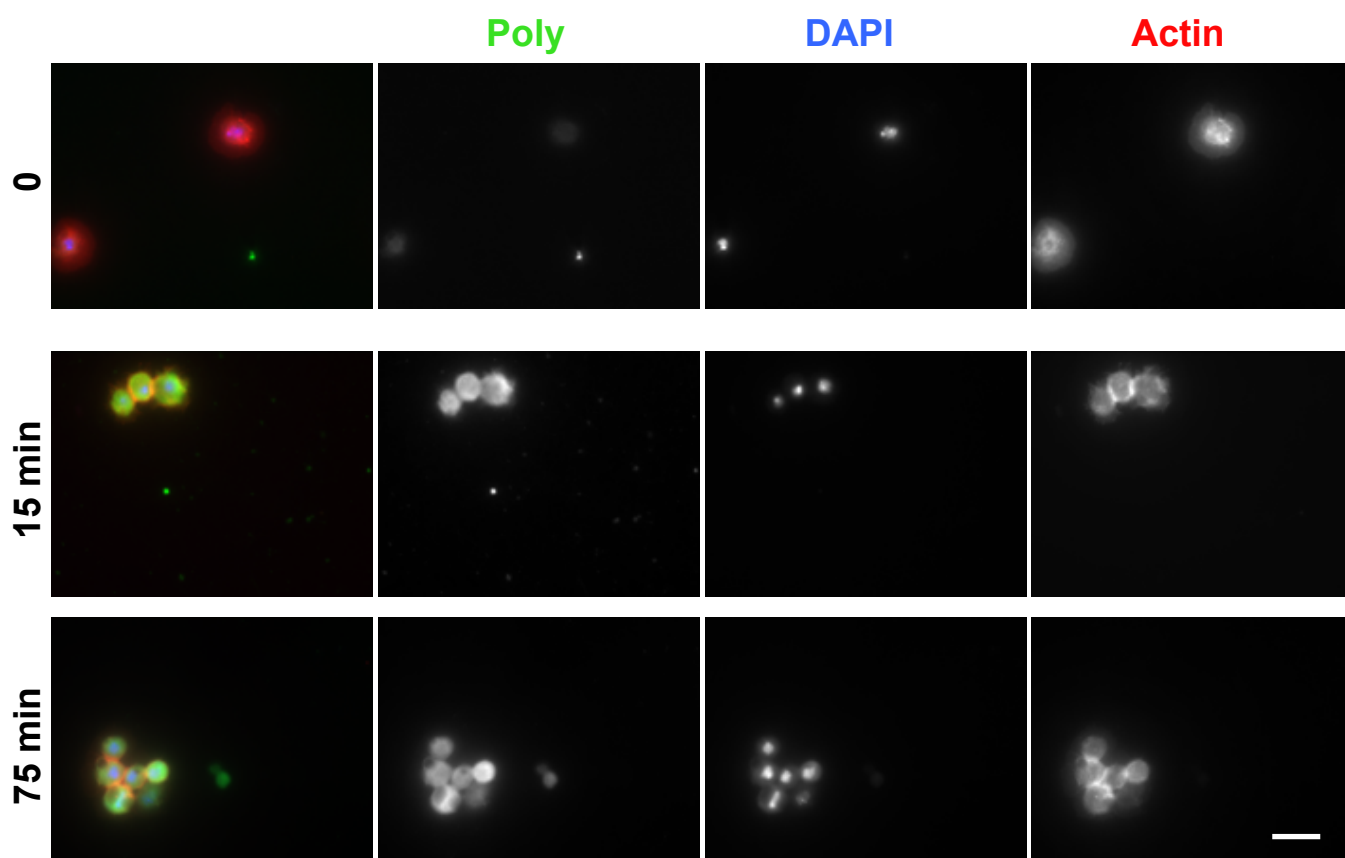

Supplemental Figure 3  
Bolukbasi, *et al.*

Supplement: Supplemental Figure 3. Poly immunostaining increases following insulin stimulation of hemocytes isolated from starved larvae [file rsob110031-s3.pdf]
